# Institutional capacity to prevent and manage research misconduct: perspectives from Kenyan research regulators

**DOI:** 10.1186/s41073-023-00132-6

**Published:** 2023-07-12

**Authors:** Edwin Were, Jepchirchir Kiplagat, Eunice Kaguiri, Rose Ayikukwei, Violet Naanyu

**Affiliations:** 1 Department of Reproductive Health, Moi University, Box 4606 -30100, Eldoret, Kenya; 2 AMPATH Research Program, Moi University and Moi Teaching and Referral Hospital, Box 4606 -30100, Eldoret, Kenya; 3 School of Arts and Social Sciences, Moi University, Box 3900 -30100, Eldoret, Kenya

**Keywords:** Prevention and management, Research misconduct, Institutional capacity, Kenya

## Abstract

**Background:**

Research misconduct i.e. fabrication, falsification, and plagiarism is associated with individual, institutional, national, and global factors. Researchers' perceptions of weak or non-existent institutional guidelines on the prevention and management of research misconduct can encourage these practices. Few countries in Africa have clear guidance on research misconduct. In Kenya, the capacity to prevent or manage research misconduct in academic and research institutions has not been documented. The objective of this study was to explore the perceptions of Kenyan research regulators on the occurrence of and institutional capacity to prevent or manage research misconduct.

**Methods:**

Interviews with open-ended questions were conducted with 27 research regulators (chairs and secretaries of ethics committees, research directors of academic and research institutions, and national regulatory bodies). Among other questions, participants were asked: (1) *How common is* research misconduct *in your view?* (2) *Does your institution have the capacity to prevent* *research misconduct?* (3) *Does your institution have the capacity to manage* *research misconduct?* Their responses were audiotaped, transcribed, and coded using NVivo software. Deductive coding covered predefined themes including perceptions on occurrence, prevention detection, investigation, and management of research misconduct. Results are presented with illustrative quotes.

**Results:**

Respondents perceived research misconduct to be *very* common among students developing thesis reports*.* Their responses suggested there was no dedicated capacity to prevent or manage research misconduct at the institutional and national levels. There were no specific national guidelines on research misconduct. At the institutional level, the only capacity/efforts mentioned were directed at reducing, detecting, and managing student plagiarism. There was no direct mention of the capacity to manage fabrication and falsification or misconduct by faculty researchers.

We recommend the development of Kenya code of conduct or research integrity guidelines that would cover misconduct**.**

**Supplementary Information:**

The online version contains supplementary material available at 10.1186/s41073-023-00132-6.

## Introduction

There is increasing interest in the integrity of the research process from conceptualization to implementation, dissemination and archiving [1]. Errant behaviors related to the handling of the research process constituting research misconduct are receiving considerable attention in the global scientific community [2, 3]. Misconduct is often defined as fabrication, falsification, and plagiarism (FFPs) [4].

Reports on research misconduct have mainly been from high-income countries such as the United States of America and the United Kingdom [5]. More recently, similar reports have been made from lower and middle-income countries such as Nigeria [6], Kenya [7], and Middle Eastern countries such as Egypt, Lebanon, and Bahrain [8]. A meta-analysis of studies in the last decade estimates that 2.9% (95% CI 2.1–3.8%) of researchers report having committed at least 1 research misconduct. In the same meta-analysis, 15.5% (95% CI 12.4–19.2%) of researchers reported having witnessed others commit at least 1 misconduct [9].

The immediate consequence of proven misconduct in published works is a retraction from the journal with its domino effect on all other works that cited the retracted literature [10]. The impact on the global scientific enterprise includes research waste, loss of public trust in the research findings, and misinformed policies that could be harmful to the public [11].

Studies report that research misconduct (RM) is associated with several factors that can be categorized as individual, institutional, national, and global factors [11–13]. At each level, some factors enable or inhibit such behavior. Individual motivators to engage in research misconduct thrive where the institutional and national structures to prevent, detect, and sanction research misconduct are perceived to be either weak or non-existent [14, 15]. It is recognized that due to an innate tendency to deviant behavior and the need to secure tenure, promotion, or fame and commercialization of research, some researchers will commit research misconduct [16]. To address the challenge posed by this small minority of researchers, institutions, national governments or ministries of higher education need to develop and disseminate research integrity oversight mechanisms and clearly defined sanctions for proven misconduct. National legal frameworks and institutional policies should underpin such research integrity oversight structures and the sanctions proposed therein [14].

Generally, the institutional and national structures that deter engagement in research misconduct are better developed in high-income countries (HIC) while studies show that in low and middle-income countries (LMICs) such structures are either weak or non-existent [17].

In Africa, few countries and institutions have prioritized the development of structures to address the threat research misconduct poses to the scientific enterprise. Among the countries with such systems is South Africa [18]. There is an emerging interest in setting up structures within the East African countries with Uganda and Kenya demonstrating early institutional efforts to address research misconduct [19, 20]. There is also an African regional effort under the aegis of the African Research Integrity Network (ARIN) to create awareness of research integrity. A paper by one of the founders of ARIN outlines the challenges associated with research misconduct in Africa and how institutions and governments in the region could address the problem [21]. There are increasing contributions of authors from Africa on this subject [20, 22, 23]. The emergence of ARIN saw the organization contribute to the hosting in Cape Town, South Africa of the 7^th^ World Conference on Research Integrity in May 2022 [24].

The goal of our study was to explore perceptions of Kenyan ethics committee leaders, top academic and research institutional managers, and leaders at the national research regulatory bodies regarding the occurrence of and institutional capacity to prevent or manage research misconduct.

## Methods

### Study design

This cross-sectional exploratory qualitative study was part of a three-phase project to develop the capacity of Moi University to prevent and manage research misconduct. This study sought to document perceptions on the occurrence of research misconduct from the perspective of members of ethics committees, the leadership of academic and research institutions, and national regulatory bodies, hereafter referred to collectively as *research regulators*. Institutions with a tripartite mission including training, research, and extension or simply, institutions of higher learning (universities) were collectively referred to as academic institutions while research institutions were those with the primary missions of research and extension. We purposely selected officials at the highest level in the administration for two reasons. Firstly, to create awareness of RM as a threat to the scientific enterprise in the institutions they lead. Secondly, we believed that participation in the in-depth interviews would stimulate them to reflect on the RM and lead to a buy into the ideas about institutionalizing prevention and management of RM. The study was done in Kenya between June and December 2018.

### Study sample

Our study population consisted of 17 human subject administrators, secretaries, and the chairpersons of Kenyan RECs, five corresponding officials of the National Scientific and Bioethics Committee of the National Commission for Science Technology and Innovation (NACOSTI), Kenya, and the Pharmacy and Poisons Board of Kenya and a purposive sample of five research directors from participating research and academic institutions.

### Recruitment of study participants

At the time of the study, there were 28 research ethics committees and 2 national regulators. Due to the relatively small number of these clusters, we targeted the recruitment of two participants from each of the 30 institutions (28 institutional ethics committees, the National Scientific and Bioethics Committee, and the Pharmacy and Poisons Board). Letters were sent to potential participants to inform them about the study in general and invite them to participate. Through telephone calls, the study coordinator made individual appointments for the face-to-face interviews at the convenience of each specific participant. The appointments were then shared with the qualitative research expert who was in charge of the data collection. Obtaining an appointment with the higher-level respondents was challenging and required multiple requests and reappointments. Due to a lack of interest in participating, non-availability, and scheduling challenges, we succeeded in interviewing a total of 27 respondents.

### Interview process

#### Research team

One colleague, RA, a qualitative research expert with a doctorate in Social Sciences and well-versed in both research ethics and qualitative research methods led the data collection exercise. The qualitative research expert worked with a team of three research assistants all with Master's level qualifications and previous experience conducting qualitative interviews. Research Assistants underwent a 3-day training on the purpose of the study and study tools.

### Data collection

An interview guide with open-ended and probes was used to collect data. It was developed specifically for the study but with themes derived from the Research Misconduct Questionnaire-Revised (RMQ-R) [25]. The interview tool is attached as Additional file 1, and additionally, the main questions asked during the interview are highlighted in the Results section. All the interviews were conducted in English and audiotaped and field notes were also made. To start the interview, all respondents were provided with a working definition of research misconduct as *“deliberate fabrication, falsification, or plagiarism in proposing, performing, or reviewing research, or in reporting research results”*. Interviews then focused on the participant’s perceptions of the occurrence of research misconduct; the current capacity to prevent, detect and manage alleged research misconduct; and facilitators and barriers to managing research misconduct in Kenya’s institutions conducting research, as a priori themes.

The majority of the interviews were carried out at the workplaces of the participants but where privacy could not be assured, the interviews happened in nearby hotels. The median duration of interviews was 34 IQR 25–46 min. The duration of interviews appeared to vary with the participants’ experiences with, and knowledge of research misconduct noted in the institutions they represented. Participants with wider experiences and knowledge shared more during the interviews. To ensure data saturation, we collected information representative of the range of experiences an perspectives relevant to the research question. No repeat interviews were done and neither were transcripts shared with respondents for comment.

### Data management and analysis

The research assistants transcribed the audio recordings verbatim into word documents. The qualitative research expert then reviewed a random sample of the transcriptions for completeness and accuracy. All transcripts were then uploaded to NVivo version 10 for coding. A codebook was deductively developed based on the *a priori* topic areas emanating from the study tool [26]. The researchers and the research assistants then reviewed the codebook and incorporated their input into the final codebook used to code all the study transcripts. Field notes augmented the data from the transcripts, as relevant. All coded data was then categorized into thematic areas in line with the study objectives: occurrence, prevention, detection, investigation, and management of research misconduct. The demographic characteristics of the respondents were summarized using descriptive statistics. Illustrative quotes were identified and presented with relevant themes.

### Reflexivity

Two issues may have influenced our review and interpretation of the interview transcripts. First, all the authors are employees of one of the academic institutions from where some of the respondents were purposively sampled. The authors are themselves researchers in this academic setting and are therefore quite conversant with the structures and capabilities therein. This lived experience likely influenced our interpretation and the thematic emphasis in the analysis of the transcripts. Secondly, three of the authors (EW, VN, and JK) are also long-serving members of the local institutional research ethics committee and one author is a member of the National Scientific and Ethics Committee (VN). These members have dealt with suspected and confirmed cases of research misconduct. It is this cumulative experience working in a research ethics committee that led to the project that is partly described in this paper and whose goal was to develop the capacity to prevent or manage research misconduct. Again, this exposure may have influenced our perspectives in interpreting and drawing conclusions from the transcripts.

## Results

We interviewed a total of 27 participants with the majority working on research ethics committees. The participant characteristics are summarized in Table 1.

**Table 1:** Respondent characteristics (*N* = 27)

Category	Sub-category	N(%)
Workplace	Research Ethics Committee (REC) Institutional leads (Research Directors) (IL) National regulator (NR)	17(63) 5 (19) 5 (19)
Position	Chairman-Research Ethics Committee (REC)	6 (22)
	Research Director (DIR)	7 (26)
	Human Subject Administrator (HSA)	5 (19)
	Research Scientist	3 (11)
	Secretary of REC	2 (7)
	Principal Science Secretary	1 (4)
	Member of REC	3 (11)
Gender	Male Female	10 (37) 17(63)
Employment Duration	Up to 5 years 5 – 10 years > 10 years	15 (56) 6 (22) 6 (22)

### Occurrence of research misconduct

To assess the occurrence of research misconduct, respondents were asked: *“How common is research misconduct in your view?”* Participants agreed that research misconduct was rampant and that this occurred mainly among students as a participant from a research ethics committee stated:

*“And the percentage is widespread..seasoned researchers in the scale of one to ten, I would give you around three. But for students on a scale of one to ten, I would give you eight (REC 11)”. *

The participants further opined that students committed research misconduct more commonly largely due to a lack of knowledge of research integrity. An institutional leader further explained that the more established researchers are more concerned about their careers while students just want to complete their studies. The participant said:

*“So to answer your question I would say I believe it is widespread among the students more than the seasoned researchers. Okay, let me say when I look at academic institutions, you know a seasoned researcher, he or she is may….very much concerned about the publication. My name outside there, I am a renowned researcher in pediatrics so I don't want to engage in such research misconduct. But if I go back now to the student, for example, my interest will be to graduate. My interest will be just to finish and satisfy my parent and the community (IL1)”.*

In the opinion of this participant, therefore, the motivation to engage in research misconduct was inversely correlated with the length of the career and the related reputational investment.

### Current institutional capacity to prevent research misconduct

The participants mentioned activities to create awareness on research integrity through seminars and presentations; provision of guidelines and strict supervision of students as mentioned by some of the participants.

“Actually, last year, we did at least two seminars on ethics, the importance of ethics, the importance to adhere to them (REC 10)”.

Training in research integrity and encouraging adherence to the principles was therefore an important prevention capacity that some participants reported.

### Capacity to detect research misconduct

Participants indicated that detection of research misconduct was multifaceted. First, the institutions depend on peer review of proposals by scientific committees. This happens mainly in research centers but some institutions of higher learning also have similar structures. After the internal scientific peer review, proposals are sent for research ethics review where there is an additional opportunity for the reviewers to raise red flags if they detect any signs of plagiarism. In institutions of higher learning use of external examiners to examine students’ theses reports also provides another layer of review where instances of plagiarism can be detected. Some institutions depend on free online plagiarism detection software but a few have custom-made proprietary software such as Turn-it-In^R^ or I Thenticate^R^ for deliberate plagiarism scan as one participant said:

*We have a licensed [plagiarism] software but I don’t know which one they use. But we are required to provide the CDs to any student who will present her thesis or proposals. So, they must submit alongside a hard copy, a CD containing a softcopy of actually what this is and then we ran through that software (REC8).*

These approaches mainly target plagiarism. Capacity to detect other forms of research misconduct that were mentioned by participants include whistleblowing. A participant said:

“*Other times when people bring in their studies, we will have probably a whistle blower, calling the secretariat to inform the secretariat about certain aspects that are not really, basically about a study that are not right (REC7)”*

It was however noted that the whistleblowing process is not formalized. Detection of fabrication and falsification mostly falls under the prepublication peer review system that is commonly at journal level.

### Capacity to investigate alleged research misconduct

There were varied practices for investigating alleged research misconduct cited by our participants including site visits by RECs to interrogate or observe research activities in the field to confirm that research is implemented per approved protocol. A participant said:

“*We have had such…questionable research practices. We have had an experience where the subcommittee of research actually had to do a site visit (REC7)”*

Such activities were commonly reported by participants from research ethics committees. The view was however supported by an institutional lead. In the cases where there is a substantive allegation, some institutions have a formalized process with a committee chaired by the deputy vice chancellor for academics which interrogates the allegation. A participant mentioned the existence of a committee in their institution but hinted that such committees were ad hoc. The participant said:

“*I know they constitute a committee which I think is chaired by the deputy vice chancellor in charge of academics but that is all I know but I would imagine that the institutions have strategies for investigations (REC8)”*

### Capacity to manage research misconduct

Overall, our respondents mainly from RECs gave an array of actions commonly taken in cases of research misconduct, although this was focused on student-plagiarized work. The actions ranged from cautioning and correcting the student and asking them to redo the work, to stopping research and even shredding data already collected. A participant from a research ethics committee said:

“*and with major changes probably, … the researcher has to work on it again then go through the process once more (REC10)*

In some circumstances, it was mentioned that student disqualification may be recommended. Ethics committees submit annual reports to the national regulator (NACOSTI) and this includes reports on research misconduct. One respondent indicated that cases of research misconduct were escalated or reported to the national research regulator as illustrated by this quote.

*“If there are issues with misconduct, they are escalated to NACOSTI. (REC7)”.*

Proven misconduct therefore is handled by a variety of actions including correction, suspension of the training program and escalation to the national regulator. The respondents, however, did not mention what actions the national regulator took on cases of misconduct reported to it.

### Barriers to management of alleged research misconduct

Several barriers were cited by our participants. We acknowledge that they are interrelated and overlapping, but, we present them separately only to highlight the unique aspects of each barrier as pointed out in the quotes.

#### Societal-level barriers

A culture of not following laws or guidelines was identified as an important societal barrier to management of alleged research misconduct as illustrate by one participant.

*We have a culture of not keeping the laws…not following laws, guidelines (REC17)*

This implied that even where there is guidance, this might not necessarily be helpful.

#### National-level barriers

At the national level, the lack of a national legal framework that defines research misconduct and provides guidelines for academic and research institutions on the prevention or management of misconduct was considered a barrier. Participants from the national regulator agreed that there was no guidance as exemplified by this quote:

“*.. I tell you something? At the national level. We don’t have research policy (NR 3)”*

This was consistent with the impression that it was unclear what action the national regulator takes on cases of research misconduct reported to it annually by institutional research and ethics committees.

#### Institutional-level barriers

The majority of barriers were institutional including inadequate financial support for research ethics, inadequate personnel for structures such as ethics committees as well as supervisors and reviewers of student research. Another barrier was the lack of related guidelines and the dissemination to inform both students and supervisors of expectations. To detect plagiarism, for example, there are dedicated software such as Turn–it–in or iThenticate but these were rarely available in academic institutions. The idea of plagiarism scans was reported to be new and only some institutions have the software as was highlighted by one participant thus:

“*It [plagiarism scans] is coming up [but]it has not picked up. We are now aware of Turn-it-in and among other soft wares that can help detect (REC10)”.*

The academic institutions also do not have any peer forum for exchanging views on research misconduct and how to manage it. The challenges of patchy capacity and lack of a common database of all past works in the various universities against which plagiarism checks can be carried out were also mentioned. A participant put it thus:

” *… because if people are using the same platform, it will be easy for me to know this work had been approved by [XX ethics committee] or had been rejected by [the same committee]. But, unfortunately the universities have not agreed on a common platform (REC3).*

Coordinated structures such as databases of previous works and dedicated antiplagiarism software would be essential for curbing plagiarism as the commonest misconduct mentioned.

#### Individual-level barriers

At the individual level, participants mentioned individual laxity and failure to optimally use structures available at their disposal. Additionally, a lack of commitment to quality research work especially through diligent guidance and supervision of students was also cited as a barrier as noted by a participant thus:

*“But then, if you look at it [student research work] there [are] gaping issues and you are like somebody else went through it for you, you just become a rubber stamping IREC (REC13)”.*

Participants were evidently pointing to the need to encourage and motivate faculty to diligently supervise student work to entrench good science and research integrity.

## Discussion

In this exploratory qualitative study involving research regulators in Kenya, our respondents perceived research misconduct to be common. Our findings are consistent with the African studies that have assessed research misconduct to be common [6, 17]. Research misconduct was viewed with a focus on plagiarism. Falsification and fabrication of research data were hardly ever mentioned as specific misconduct. Nearly all responses referred to addressing plagiarism, potentially indicating that other forms of research misconduct are neglected. Further, there was a perception that misconduct, specifically, plagiarism was a student problem. Participants did not point out that faculty can also commit research misconduct. Faculty are at risk of succumbing to individual factors associated with the commission of research misconduct due to the pressure to publish for promotion [15]. In this respect, the apparent focus on students to the exclusion of later career researchers is concerning and points to an awareness and capacity gap.

Regarding falsification and fabrication, identification requires much greater sophistication in terms of critical appraisal of the scientific literature and being alert to subtle discrepancies that raise red flags about the possibility of misconduct. Detection or suspicion of fabrication and falsification also requires a strong culture of responsible conduct of research (RCR) [24, 27] among members of research teams as well as structures for researchers to be able to report any suspicious behavior for requisite intervention [28]. It also requires robust internal and external peer review processes before and after the submission of a paper to the journals [29] to be able to pick out falsification and or fabrication of data. More recently, there is a move towards Open Science whereby study protocols including analysis plans are published ahead of study dissemination and once the data collection and cleaning is completed, data is locked and made available publicly, allowing other scientists to scrutinize the data whenever queries on research outcomes arise. This concept was adopted and incorporated into the Hong Kong Principles on rewarding researchers [30]. Respondents mentioned some internal peer review activities though the role of Open science was not mentioned.

### Capacity to prevent misconduct is informal and uncoordinated

Prevention of misconduct involves awareness creation about misconduct and its consequences, training in the broad concepts of responsible conduct of research (RCR) and capacity to detect misconduct when it occurs [23]. Indeed, it is part of RCR to report any potential misconduct for investigation and determination.

Although plagiarism detection software exists, this study identified that few of the institutions, both academic and research, had subscribed to this software and required students or faculty researchers to provide similarity indices for their works before academic assessment or peer review, respectively. Considering the frequent occurrence of plagiarism and the ease with which it can be detected using dedicated software, the perception that many institutions did not have anti-plagiarism software points to a significant missed opportunity to foster research integrity and prevent misconduct.

### Managing research misconduct is beyond the mandate of RECs and is hampered by lack of guidelines

To manage allegations of misconduct fairly, it is essential to develop mechanisms for investigating allegations, determining outcomes of investigations and sanctioning proven perpetrators. Our study identified lack of such structures, with some institutions using human resource policies to inform research misconduct management process. Given the background of perceived fairly common occurrence of research misconduct in low and middle-income economies [6, 7, 17], our findings point to a lack of capacity. Other authors have also underscored the need for a concerted effort to develop institutional and national guidelines to address research misconduct [29]. Such national guidelines are among the structures recommended for managing research misconduct and which were reported to be missing by our respondents [31].

Among the perceived capabilities mentioned by respondents was the role of RECs in management of research misconduct. RECs, also called institutional review boards (IRBs), have the core mandate of promoting and safeguarding the welfare and safety of research participants. Additionally, RECs are mandated to monitor the conduct of approved research to ensure adherence to approved protocols. While many RECs achieve their first mandate quite satisfactorily, the second is more challenging and is generally poorly implemented across many RECs due to a lack of capacity, especially where many research proposals are reviewed and approved [32, 33]. RECs collaborate with the researchers to adhere to the principles of RCR for the safety and well-being of research participants.

On the contrary, when a researcher commits research misconduct, a deliberate subversion of the principles of RCR, the processing of allegations of misconduct is a quasi-legal and adversarial proceeding that is outside the mandate and capacity of a REC. In the US, for example, while RECs/ IRBs may have a role in promoting RCR and, in whistleblowing in cases of alleged research misconduct, the task of managing research misconduct is the mandate of a research integrity oversight office that is designed and empowered to carry out adversarial proceedings for any alleged case of research misconduct [34]. Moreover, the consequences of proven misconduct may include career reputational damage and penal sanctions [35], all of which can only be implemented if underpinned by national legal frameworks. A perception that RECs/ IRBs can be depended on for the management of cases of alleged misconduct, therefore, appears misinformed.

### Strength and limitations

To our knowledge, this exploratory qualitative survey is the first of its kind to explore the perceived capacity existing within Kenyan research and higher education institutions to prevent and or manage research misconduct. The study had one important limitation. The respondents appeared to have varying definitions of research misconduct. Such variations in the definition of research misconduct have been reported by other researchers [36]. Some respondents appeared to confuse research misconduct with the much broader concept of academic misconduct. To achieve consensus, the definition of research misconduct was provided to the respondents. It is possible that the variance in definition affected the respondents' views on the scope of research misconduct leading to the apparent focus on students.

We conclude that research misconduct occurs in Kenyan institutions. However, the institutions do not have dedicated capacity to prevent or manage misconduct, a situation that is contributed to by the lack of national guidelines on research misconduct. We recommend the development of Kenya code of conduct or research integrity guidelines that would cover research misconduct. Additionally, efforts should be made to enhance awareness of research misconduct beyond plagiarism and for all researchers. Investment in and mandatory use of antiplagiarism software would be a simple but productive starting point.

## Supplementary Information

**Additional file 1.** Key Informat Interview Guide.

## Data Availability

Raw data has been provided to the editors and reviewers during peer review.

## Abbreviations

ARIN: African Research Integrity Network
DIR: Director
FFP: Fabrication, Falsification & Plagiarism
HIC: High-Income countries
HAS: Human subject administrator
IRB: Institutional Review Board
IQR: Interquartile range
LMIC: Lower Middle-income countries
MTRH/MU IREC: Moi Teaching and Referral Hospital / Moi University Institutional Research and Ethics committee
NACOSTI: National Commission for Science Technology and Innovation
QRP: Questionable Research Practices
RA: Research Assistant
RCR: Responsible Conduct of Research
REC: Research Ethics Committee
RMQ-R: Research Misconduct Questionnaire-Revised
WCRI: World Conference on Research Integrity
