# Supplementary material for: Institutional capacity to prevent and manage research misconduct: perspectives from Kenyan research regulators
Source: Res Integr Peer Rev. 2023 Jul 12;8:8. doi: 10.1186/s41073-023-00132-6 (PMC10337100; doi:10.1186/s41073-023-00132-6)
Supplement: Supplementary file 1 — Additional file 1. Key Informat Interview Guide. [file 41073_2023_132_MOESM1_ESM.pdf]

Developing Capacity of Moi Teaching and Referral Hospital / Moi University Institutional Research Ethics Committee (MTRH/MU IREC), Kenya to Prevent and Manage Research Misconduct

AIM 2: Explore the perceptions on capacity to detect and manage research misconduct and the perceived critical components of a model framework for managing research misconduct

Target Population: REC officials (chair, Secretary, Administrator), NACOSTI, PPB.

Introduction

I am ….. from Moi University. We are collecting information about Research Misconduct defined as "as deliberate fabrication, falsification, or plagiarism in proposing, performing, or reviewing research, or in reporting research results". RM is an important emerging threat to the research enterprise locally and globally. We wish to ask you a few questions to help us understand how RM can be managed in Kenya and if the requisite capacity to manage RM is available in Kenyan institutions that carry out research. You are being invited to participate because you work in an organization that regulates research in Kenya. Your participation is voluntary. If you accept to participate, we request that you permit us to audio-tape the discussion so that we can be as accurate as possible in our efforts to capture the information you provide. The information you provide shall be confidential and no information that can identify you as a person will be disseminated. There are no significant risks in participating as we are only seeking your opinion. There are no right or wrong answers. There are no benefits to you from the study but the information may help design a framework for managing RM that can be adapted across the country. The interview may take about 30 minutes of your time. If you accept to participate may we begin?

1. Socio-demographic data
2. Position of respondent
3. Comment on the definition of Research Misconduct (RM)
4. Causes of RM –

a) Individual factors
b) Institutional factors
c) National factors
d) Global factors
e) Other factors

5. How common is RM in your view?
6. Does your institution have capacity to Prevent RM? Explain (probe: plagiarism, fabrication, falsification, other questionable research practices)
7. Does your institution have capacity to Detect RM? Explain (probe: plagiarism, fabrication, falsification, other questionable research practices)
8. Does your institution have capacity to Investigate RM? Explain (probe: plagiarism, fabrication, falsification, other questionable research practices)

9. Does your institution have capacity to Manage RM? Explain (probe: plagiarism, fabrication, falsification, other questionable research practices)

10. Barriers to Management of alleged RM

11. Ideal Structure of the Institutional Framework to Manage alleged RM

a) Preventing RM

b) Detecting RM

c) Investigating RM

d) Managing a proven case of RM

Any other final thoughts?

Thank you for participating
